# Interleukin 17 B regulates colonic myeloid cell infiltration in a mouse model of DSS-induced colitis

**DOI:** 10.3389/fimmu.2023.1055256

**Published:** 2023-02-06

**Authors:** Xiaomin Zhang, Xiaokai Zhang, Xiaomei Song, Chuanying Xiang, Chunmei He, Yu Xie, Yangyang Zhou, Ning Wang, Gang Guo, Weijun Zhang, Yan Li, Kaiyun Liu, Quanming Zou, Hong Guo, Yun Shi

**Affiliations:** ^1^ Institute of Biopharmaceutical Research, West China Hospital, Sichuan University, Chengdu, Sichuan, China; ^2^ National Engineering Research Center of Immunological Products, Department of Microbiology and Biochemical Pharmacy, College of Pharmacy, Third Military Medical University, Chongqing, China; ^3^ Department of Gastroenterology, Chongqing General Hospital, Chongqing, China; ^4^ Department of Gastroenterology, Southwest Hospital, Third Military Medical University, Chongqing, China

**Keywords:** inflammatory bowel disease, neutrophils, intestinal macrophages, myeloid Cells, S100A9, single cell RNAseq

## Abstract

Cytokines play vital roles in the pathogenesis of inflammatory bowel disease. IL17B is protective in the development of colitis. However, how IL17B regulates intestinal inflammation and what cells are regulated by IL17B is still unknown. Here, we aimed to illustrate the IL17B dependent cellular and molecular changes in colon tissue in a mouse colitis model. The results showed that IL17B expression in colon tissues was elevated in inflamed tissues than non-inflamed tissues of IBD patients. Wild type (WT) and *Il17b* deficient (*Il17b* ^-/-^) mice were given 2.5% dextran sodium sulfate (DSS) water, and in some case, *Il17b* ^-/-^ mice were treated with recombinant mouse IL17B. IL17B deficiency resulted in severe DSS-induced colitis with exaggerated weight loss, shorter colon length, and elevated proinflammatory cytokines in colon. Reconstitution of *Il17b* ^-/-^ mice with recombinant IL17B alleviated the severity of DSS-induced colitis. Single cell transcriptional analyses of CD45^+^ immune cells in colonic lamina propria revealed that loss of IL17B resulted in an increased neutrophil infiltration and enhanced inflammatory cytokines in intestinal macrophages in colitis, which were confirmed by real-time PCR and flow cytometry. IL17B treatment also inhibited lipopolysaccharide-induced inflammation in bone marrow-derived macrophages and mice. IL17B inhibits colitis by regulating colonic myeloid cell response. It might represent a novel potential therapeutic approach to treat the colitis.

## Introduction

Inflammatory bowel disease (IBD), including ulcerative colitis and Crohn’s disease, is a chronic inflammatory disease of the gastrointestinal tract with increased morbidity and negatively influence the life quality (1). IBD development is involving dysregulation of the genetic, environmental, microbiota, and immune responses (2). Disruption of the intestinal epithelial barrier triggers the immune reorganization of commensal bacteria and induces an inflammation response characterized by the production of inflammatory cytokines, including tumor necrosis factor (TNF), interleukin 1 beta (IL1B), IL6, IL12, and IL18, which are responsible for IBD pathogenesis (3).

Cytokines play an important role in the development of IBD by regulating the recruitment and differentiation of colonic immune cells (3). IL17 families containing IL17A, IL17B, IL17C, IL17D, IL17E (IL25), and IL7F are all reported to be involved in IBD (4, 5). Among these, the role of IL17A, IL17F, IL17C, and IL17E in colitis and their mechanisms have been thoroughly investigated (4–6), whereas IL17B is less studied. IL17B is expressed by colonic epithelial cells and functions through the receptor IL17RA and IL17RB, which is also the receptor for IL25. IL17B is reported to be protective in dextran sulfate sodium (DSS)-induced colitis mice model, since deficiency of IL17B results in increased susceptibility to colonic inflammation (6). The protective role of IL17B in colitis is assumed to competitively inhibit IL25-driven colon inflammation (6). However, the role of IL17B in IBD patients and the detailed mechanisms of IL17B in inhibition of inflammation have not been illustrated.

The immune response in colon lamina propria is important for IBD progress. The misregulation of myeloid cell response, T cell response, and B cell response were all involved in IBD pathogenesis. How IL17B affects the landscape of immune cells in colon tissue is still unknown.

In this study, we used single cell RNA sequencing (scRNAseq) to reveal that IL17B deficiency led to an altered immune cell landscape in DSS-induced colitis mouse model. IL17B exerts its protective role on colitis by inhibiting neutrophils infiltration, and suppressing proinflammatory cytokines production in intestinal macrophages in colon. Administration of IL17B ameliorates colitis in mice. IL17B is a novel inhibitory cytokine which could be potentially serve as biomarkers and protective new targets harnessed for colitis therapy.

## Materials and methods

### Clinical samples

Human biopsy specimens of colon mucosal tissues were collected from a total of 23 IBD patients with a clinical diagnosis of UC (n = 10) and CD (n = 13). The paired active endoscopic inflamed tissues and non-inflamed tissues were obtained through endoscopic colonic biopsy conducted by the Gastroenterology Department at Chongqing General Hospital. Clinical samples collection was approved by the Ethics Committee at Chongqing General Hospital (Approval number: KY S2022-023-01). Informed consent was obtained from all patients. The clinical information of the patients are list in **Supplementary Table 1**.

### Real-time PCR

The total RNA was isolated from colonic tissues or cells using RNApure Tissue & Cell Kit (CoWin Biosciences, Beijing, China) and reverse transcribed to cDNA with PrimeScript™ RT reagent Kit (Takara Biotechnology, Dalian, China). Real-time PCR was performed using SYBR Green Master Mix (Takara Biotechnology) on CFX96 system (Biorad, CA, USA) with specific primers listed in **Supplementary Table 2**. The expression level of the relative genes was normalized relative to levels of the housekeeping gene *Actb* and calculated using 2^−ΔΔCt^ method.

### Mice

Six-to eight-week-old female C57BL/6 mice were purchased from Beijing HFK Bioscience Limited Company (Beijing, China). IL17B deficient (*Il17b* ^-/-^) mice (C57BL/6-IL17Btm1cyageI) were constructed by Cyagen Biosciences (Suzhou, Jiangsu, China) using CRISPR/Cas9 technology by deleting the exon 2 of *Il17b*. Weight, sex, and age-matched mice were used in all studies. Cohoused WT mice were used as controls for all experiments. The animal studies were approved by the Institutional Animal Care and Treatment Committee of West China Hospital, Sichuan University (Approval number: 2020372A), and carried out according to the National Institutes of Health Guide for the Care and Use of Laboratory Animals.

### DSS-induced colitis

Mice were fed with 2.5% (w/v) DSS (36,000–50,000 daltons; MP Biomedicals, Solon, OH, USA) in drinking water for 6 days and changed back to water. Mice were weighed daily. The body weight loss was calculated as the percentage of the starting weight. Colon length was measured.

### Histopathology

The intermediate colon tissue was cut and fixed immediately in 4% formalin and embedded in paraffin. Sections were cut in 5 μm thick, stained with hematoxylin-eosin and observed by a light microscope. Histological scoring were evaluated as the sum of two parameters according to the following criterion. Epithelium: 0 = normal; 1 = loss of goblet cells; 2 = loss of goblet cells in large areas; 3 = loss of crypts; 4 = loss of crypts in large areas; Inflammatory cell infiltration: 0 = no infiltrate; 1 = infiltrate around crypt basis; 2 = infiltration in the muscularis mucosa; 3 = extensive infiltration in the muscularis mucosa with edema; and 4 = infiltration of the submucosa.

### Colon cytokine detection by ELISA

Colons were weighed and homogenated with 1 mL PBS. The supernatants was taken to measure cytokines by ELISA and normalized to the colon weight.

### ELISA

TNF, IL1B, IL6, and CXCL1 in colon homogenates or serum were measured using mouse TNF ELISA kit, mouse IL6 ELISA kit, mouse IL1B ELISA kit (Biolegend, San Diego, CA, USA) and mouse CXCL1 ELISA Kit (Invitrogen, Carlsbad, CA, USA) accordingly to the manufacturer’s instruction.

### Colonic immune cells isolation

Colons tissue were incubated in digestion solution of Ca2^+^ and Mg2^+^ free PBS with 5 mM EDTA and 1 mM dithiothreitol to remove colon epithelial cells. Remaining tissue was digested using PBS with Ca2^+^ and Mg2^+^ supplemented with 0.3 mg/ml collagenase VIII (Sigma-Aldrich, St. Louis, MO, USA) and 0.3 mg/ml DNase I (Dingguo Changsheng, China) and 5% fetal bovine serum (FBS, Gibco, Melbourne, VIC, Australia). The cell suspension was passed through a 70 μm and a 40 μm cell strainer sequentially to get the single cells.

### Single-cell RNA sequencing

Single-cell capturing CD45^+^ immune cells and downstream library construction were performed on BD Rhapsody system (BD Biosciences, New Jersey, USA) following the manufacturer’s instruction. Briefly, single cell suspensions of each colonic lamina propria sample from 2 WT and 2 *Il17b* ^-/-^ colitic mice were stained with anti-mouse CD45-biotin (30-F11, BD Biosciences) and a separate sample tag (sample tag 1 to 4) using the mouse immune Single-Cell Multiplexing Kit (BD Biosciences). Then CD45^+^ immune cells were sorted using Streptavidin microbeads Kit (Miltenyi Biotech, Bergisch Gladbach, Germany). Viability of cell was > 85% evaluated by trypan blue exclusion. Sample tag labeled single cell of four samples was pooled together (~20,000 cells) to load onto a BD Rhapsody Cartridge for single-cell mRNA capture using BD Rhapsody Cartridge reagent Kit (BD Biosciences). Beads with captured mRNA then were retrieved from cartridge into a single tube and cDNA were synthesis on beads using BD Rhapsody cDNA kit. The cDNA was split to construct the mRNA whole transcriptome analysis (WTA) library and sample tag library using BD Rhapsody WTA Amplification Kit following the BD Rhapsody protocol. The libraries were sequenced on Illumina nova6000.

### ScRNAseq data analysis

The sequenced data were processed *via* BD Rhapsody analysis pipeline on Seven Bridges (SBG, https://www.sevenbridges.com). Briefly, we run BD Rhapsody WTA analysis pipeline to analyze WTA raw reads with the genome reference of GRCm38 and sample multiplex selected of “Single-Cell Multiplex Kit-Mouse”. The output files of molecule counts of each sample were loaded on SeqGeq v1.8 software (BD biosciences) for downstream bioinformatics analysis. First, four samples were concatenated to one sample. We did the dimensionality reduction using the Seurat plugin in SeqGeq with default parameter to get the clusters and differentially expressed genes (DEGs) for defining the clusters. Uniform Manifold Approximation and Projection (UMAP) was used to show the clusters. The DEGs in each cluster between WT and *Il17b^-^* ^/-^ mice were calculated in SeqGeq by statistics with a *P* value < 0.05 and Log_2_ Fold-Change > 0.25 for upregulated genes. The heatmap was also created in SeqGeq. Gene enrichment analysis was performed using Metascape webtool (https://www.metascape.org) (7). For subclustering, all the cluster of myeloid cells were gated as a group and do the reclustering using plugin Seurat in SeqGeq again as above.

### Flow cytometry

The colonic lamina propria cells were blocked with rat serum then stained with live/dead dye FVS700 (BD Biosciences) and the fluorophore-conjugated surface marker antibodies BV605-conjugated anti-CD45 (BD Biosciences), anti-CD11B, and anti-Ly6G antibody (Biolegend). After surface staining, cells was fixed and permeabilized with Foxp3/transcription factor staining buffer set (Thermo Fisher Scientific, MA, USA) followed by staining with AF647-anti S100A9 (BD Biosciences). Cells was acquired on BD FACSCanto™ II (BD Biosciences) and data were analyzed with FlowJo^®^ ver. 10 (BD Biosciences).

### Recombinant IL17B administration

*Il17b* ^-/-^ mice and WT mice were induced colitis by feeding 2.5% or 3% DSS water respectively, and intraperitoneally (I.P.) injected with 0.5 μg of mouse IL17B (R&D Systems, Minneapolis, MN, USA) or PBS. The treatment was performed 1 hour before the initial DSS administration and repeated every 48 hours for 4 times. Severity of colitis was assessed as described above.

### Bone marrow derived macrophages (BMDM)

Bone marrow cells was extracted from the femur of mice and cultured in DMEM supplemented with 25 ng/mL murine macrophage colony-stimulating factor (R&D Systems). On day 7, BMDM were stimulated with lipopolysaccharides (LPS, 10 ng/ml, Sigma-Aldrich) and IL17B (50 ng, 200 ng/ml) for 2 hours and mRNA of cytokines in BMDM were detected by real-time PCR.

### Bulk RNAseq

BMDM were treated with IL17B (200 ng/ml) or PBS as a control (3 samples for each treatment) for 6 hours and total RNA was extracted using Trizol (Invitrogen, Carlsbad, CA, USA). Total RNA was quantified using a Nano Drop and Agilent 2100 bioanalyzer (Thermo Fisher Scientific) and used to prepare sequencing library and sequenced on BGIseq500 platform (BGI-Shenzhen, China).

### Data processing of RNAseq

The sequencing data was filtered with SOAPnuke (v1.5.2). Clean reads were obtained and mapped to mouse genome GRCm38 using HISAT2 (v2.0.4). Bowtie2 (v2.2.5) was applied to align the clean reads to the reference coding gene set, then expression level of gene was calculated by RSEM (v1.2.12). Essentially, differential expression analysis was performed using the DESeq2 (v1. 4.5) with Q value ≤0.05. Gene enrichment analysis was performed using Metascape webtool.

### LPS sepsis model

WT mice were I.P. treated with LPS (50 μg/kg) plus IL17B (50 μg/kg) or PBS. Two hours later, blood and colon tissue was taken for cytokines detection.

### Statistical analysis

Data are expressed as mean ± standard error (SD). Statistical analysis was performed using the GraphPad Prism version 5.00 (Graphpad software, La Jolla, CA, USA). Body weight loss was evaluated by 2 way ANOVA analysis with Sidak`s multiple comparisons test. The paired or unpaired *t* test was used to assess statistical differences between two matched or unmatched samples. * *P* < 0.05; ** *P <*0.01; *** *P* < 0.001; **** *P* < 0.0001. All experiments were repeated at least twice except scRNAseq and bulk RNAseq.

## Results

### IL17B is elevated in the colon of patients with IBD

**Figure 1:**
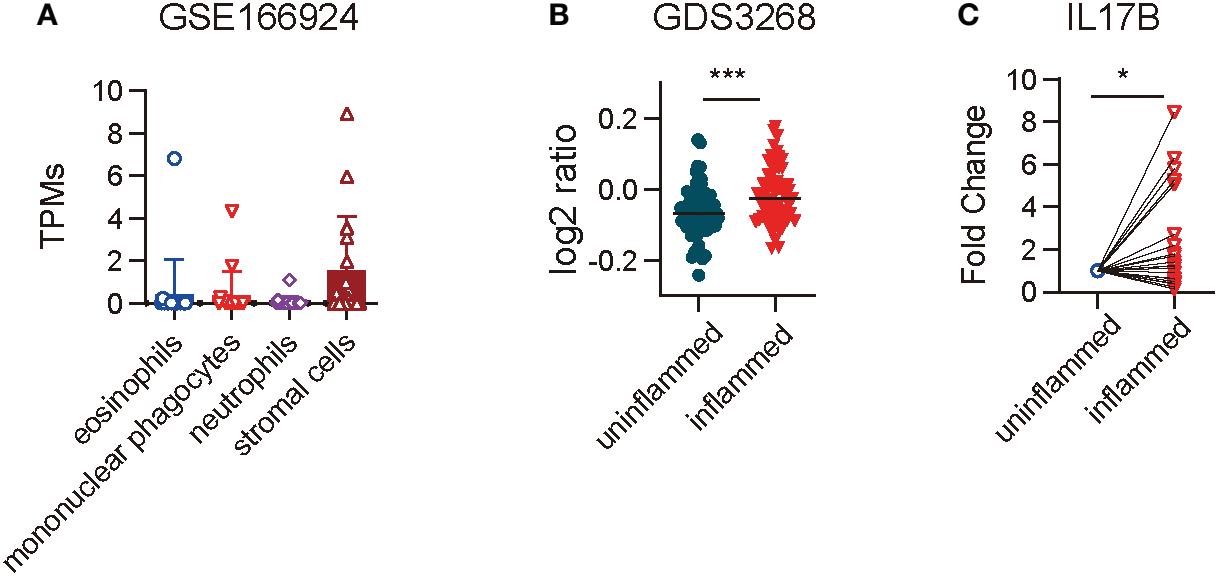
IL17B expression was increased in the colon tissue of IBD patients. **(A)** IL17B expression in different cell types isolated from endoscopic biopsies of patients with IBD in a RNAseq dataset GSE166924. n=17 **(B)** Quantification of IL17B expression of colon uninflamed tissues (n=66) and inflamed tissues (n=63) from UC patients with a microarray data set GDS3268. ***P < 0.001 determined by student t test. **(C)** IL17B expression in paired colon inflamed tissues and non-inflamed tissues from 23 IBD patients was detected by real-time PCR. Data represent mean ± SD, *P < 0.05; determined by paired student *t* test.

IL17B expression in mouse colitis model has been reported (6), but its expression in IBD patients is still unknown. First we analyzed the RNAseq data of GSE166924 (8) and a microarray dataset of GDS3268 (9) of IBD patients from National Center for Biotechnology Information (NCBI) Gene Expression Omnibus (GEO) data sets. The results showed that IL17B mainly expressed in stromal cells in human colon tissue (**Figure 1A**). Further analysis of RNAseq data from colon epithelial biopsies of UC patients showed an increase of IL17B in inflamed tissue of IBD when compared with unaffected regions (**Figure 1B**). To confirm the results, we then collected colon biopsies from IBD patients and detected IL17B expression by real-time PCR. Compared to uninflamed region, samples of patients with active inflammation had a significant increase of IL17B mRNA level compared to the paired non-inflamed colon tissue (**Figure 1C**). These data indicate that IBD patients have increased IL17B expression in colon tissue.

### IL17B deficiency exacerbates DSS-induced colitis

**Figure 2:**
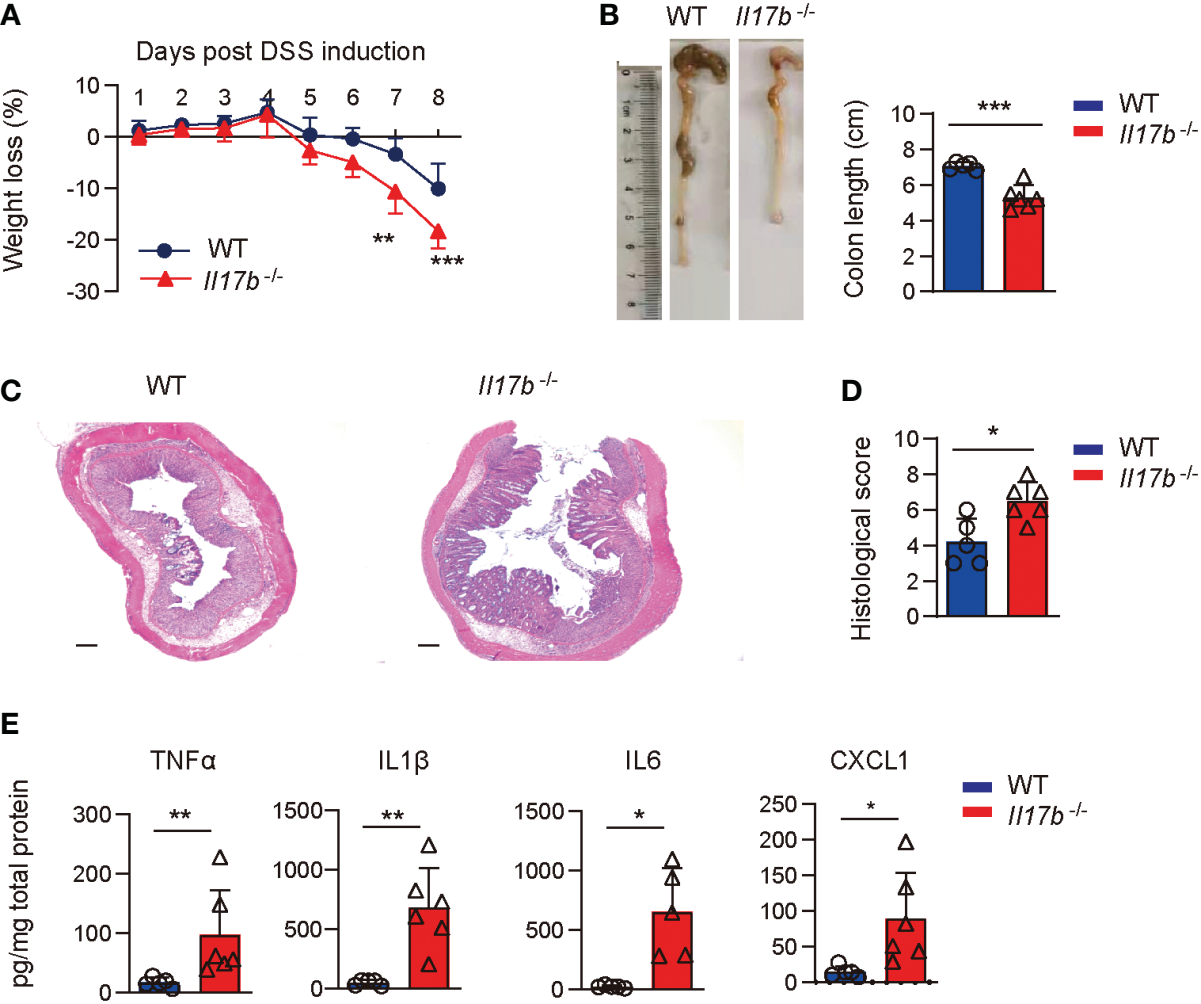
IL17B deficiency results in exacerbated DSS-induced colitis. WT and *Il17b* ^-/-^ mice were fed with 2.5% DSS water for 6 days. n = 5 - 6 mice/group. **(A)** Body weights were measured daily and body weight loss relative to initial weight were calculated. Data are presented as mean ± SD. **P < 0.01, ***P < 0.001, evaluated by 2way ANOVA analysis with Sidak`s multiple comparisons test. **(B)** Colons were removed at day 8 and the colon length was measured. **(C)** Representative H&E staining of colon tissues. Scale bar: 200 μm. **(D)** Quantification of histological score from colonic sections in **(C, E)** The supernatants of colon tissue homogenates were assayed for cytokine by ELISA. For **(B, C)**, data are mean ± SD and are representative of at least 3 independent experiments. *P < 0.05, **P < 0.01, ***P < 0.001, evaluated by unpaired *t* test.

To explore the mechanisms of IL17B in the development of colitis, we created a mouse strain with a targeted deletion of exon 2 of IL17B by CRISPR/Cas-mediated genome engineering and confirmed the deletion of IL17B gene (**Supplementary Figures 1A, B**). We then induced the colitis with DSS in WT and *Il17b* ^-/-^ mice. We found that *Il17b* ^-/-^ mice lost more weight (**Figure 2A**) and colon lengths were consistently shorter than their WT counterparts upon DSS treatment (**Figure 2B**). H-E staining of the colon tissues showed greater inflammatory cells infiltration and epithelial cells destruction in *Il17b* ^-/-^ colitis mice (**Figures 2C, D**).We measured the levels of the inflammatory cytokines in colon homogenates at day 8 of colitis. TNF, IL1B, and IL6 were increased in *Il17b* ^-/-^ mice compared with WT in colitis in both protein (**Figure 2E**) and mRNA levels (**Supplementary Figure 1C**). However, mRNA expression of Il17, Il22, Ifnγ, and Il25 were not significantly changed (P < 0.05, **Supplementary Figure 1C**). Therefore, these results indicate that IL17B can ameliorate colitis in mice.

### Supplement of IL17B alleviates DSS-induced colitis

**Figure 3:**
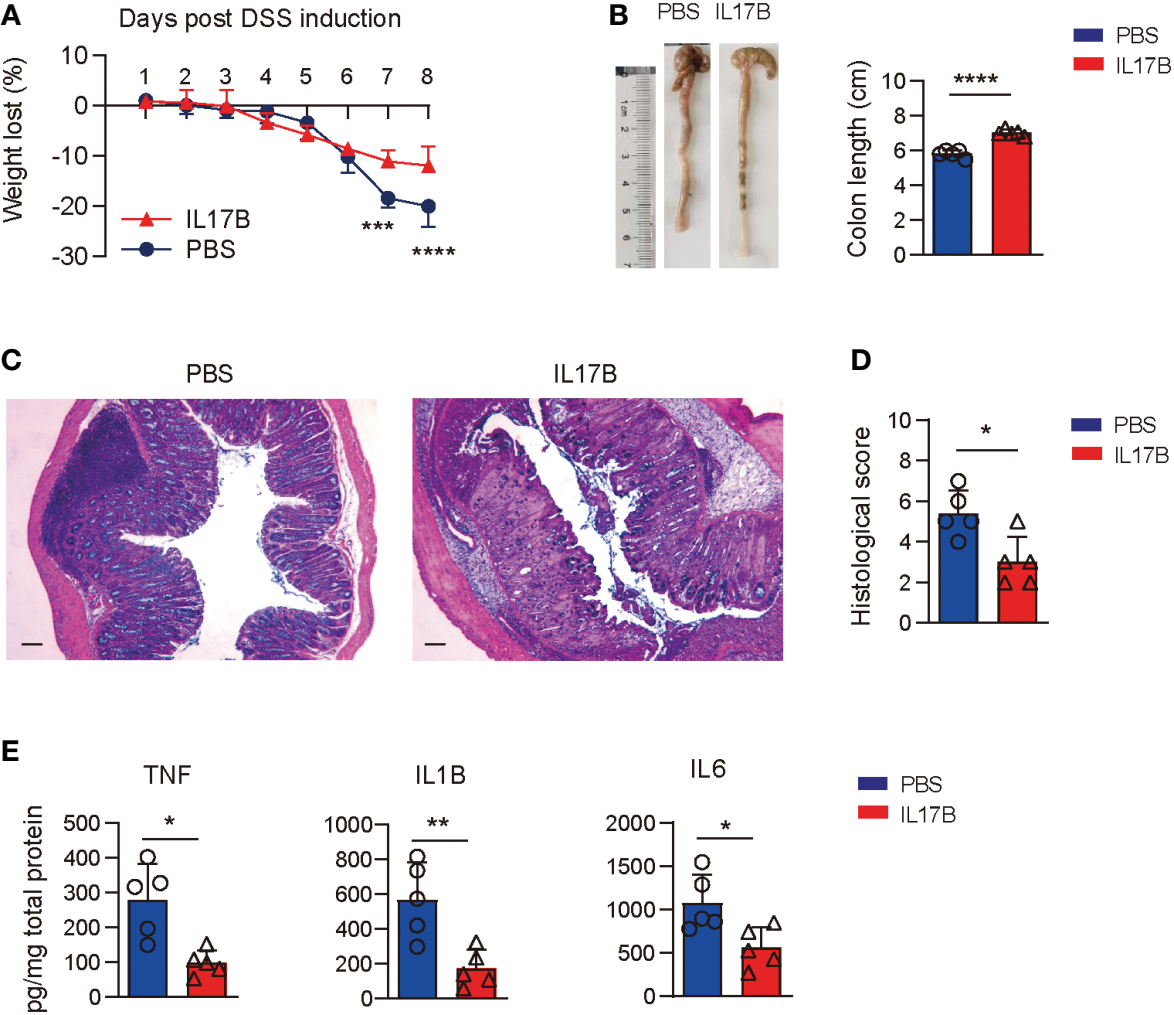
Rescue of IL17B alleviates the severity of colitis. *Il17b* ^-/-^ mice were induced colitis by feeding 2.5% DSS water and intraperitoneally (I.P.) injected with 0.5 μg of mouse IL17B (n = 4) or PBS (n=3). The treatment was performed 1 hour before the initial DSS administration and repeated every 48 hours for 4 times. **(A)** Body weight loss was measured daily. ***P < 0.001, ****P < 0.0001, evaluated by 2 way ANOVA analysis with Sidak`s multiple comparisons test. **(B)** The colon lengths were measured on day 8. **(C)** Representative H&E staining of colon tissues. Scale bar: 200 μm. **(D)** Quantification of histological score from colonic sections in **(C, E)** The supernatants of colon tissue homogenates were assayed for cytokine by ELISA. Data are mean ± SD. *P < 0.05, **P < 0.01, ****P < 0.0001, evaluated by unpaired *t* test.

To further confirm the protective role of IL17B in colitis, we treated *Il17b* ^-/-^ mice with recombinant mouse IL17B to see if it could reduce the colitis severity. We found that colitic mice treated with IL17B I.P. had reduced weight loss (**Figure 3A**) and longer colons compared those treated with PBS (**Figure 3B**). The same rescue effect of IL17B on DSS colits was abserved in WT mice (**Supplementary Figure 2**). histopathological analysis of colon tissues revealed that IL17B alleviate the inflammatory cells infiltration and epithelial cells destruction in *Il17b* ^-/-^ colitis mice (**Figures 3C, D**)Further, treatment of IL17B significantly reduced TNF, IL1B, and IL6 expression in colon tissue in *Il17b* ^-/-^ colitic mice (**Figure 3E**).These data confirm that IL17B protects DSS-induced colitis.

### IL17B deficiency leads to an altered colonic immune cells composition in colitis

**Figure 4:**
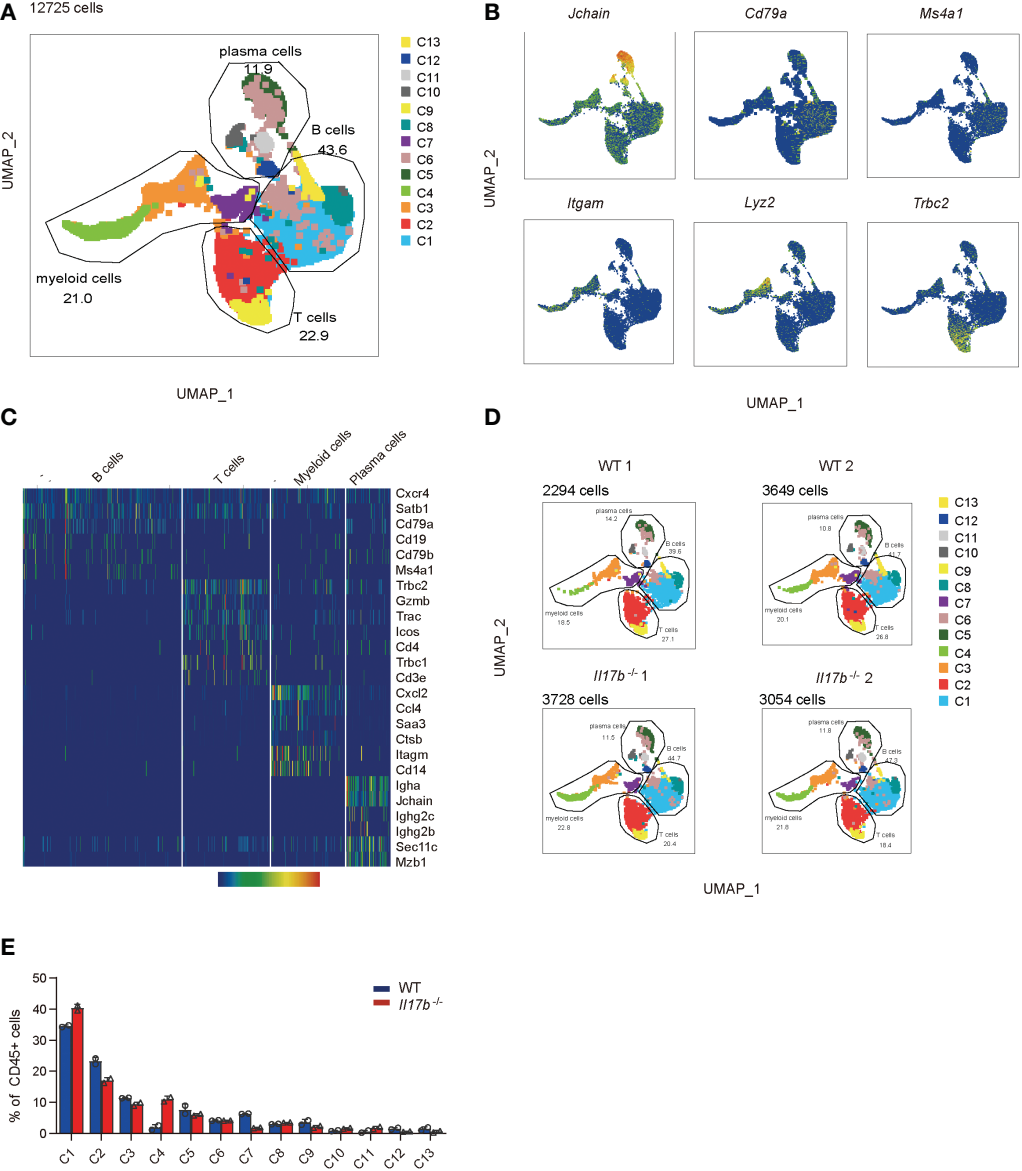
Single cell transcriptome analysis of colon lamina propria immune cells in colitis in WT and *Il17b* ^-/-^ mice. Two WT and two *Il17b* ^-/-^ mice were fed with 2.5% DSS water to induce colitis. Lamina propria CD45^+^ immune cells were sorted by magnetic activated cell sorting and processed for scRNAseq. **(A)** UMAP plot shows the clustering of 12725 immune cells, colored by cell subset and cell types. **(B)** UMAP plots displaying marker genes expression for cell cluster. **(C)** Heatmap showing specific marker genes for major cell types. **(D)** UMAPs plots showing a comparison of the CD45^+^ cell clusters distribution in WT and *Il17b* ^-/-^ colitic mice. **(E)** Percentage of each cell cluster in CD45^+^ cells in WT and *Il17b* ^-/-^ colitic mice.

Intestinal immune cells including T cells, B cells, and myeloid cells are all reported to be important in colitis development. Further we sorted CD45^+^ immune cells from mouse colonic lamina propria on day 8 after colitis induction and performed single cell RNA sequencing (scRNAseq) to find out the effect of IL17B on colonic immune response in colitis. To avoid bath effects, we stained cells from each mouse of 2 WT and 2 *Il17b* ^-/-^ colitis mice with sample tags and pooled them together for downstream process using BD Rhapsody system. After quality control, we obtained 15584 cells with an average of 354.22 genes per cells profiled, resulting in a total of 21702 mouse genes detected in all cells (**Supplementary Table 3**). Unbiased uniform manifold approximation and projection (UMAP) clustering identified 14 cell population, including CD45^+^ immune cells expressing Ptprc (encoding CD45) and stromal cells with no express of Ptprc (**Supplementary Figure 3**), which were considered as contaminating non-immune cells and excluded from downstream analyses. Further, we focused on CD45^+^ immune cells (12725 cells) and did a UMAP clustering resulting in 13 cell clusters (**Figure 4A** and **Supplementary Table 4**). These clusters were characterized by identifying signature genes for each cluster and were identified to belong to 4 cell types (**Figures 4A–C**). A total of 4 B cell clusters (C1, C6, C8, and C13) were characterized by expression of Ms4a1 (encoding CD20), Cd19, and Cd79b; 3 clusters of plasma cells (C5, C10, and C11) were characterized by expression of Jchain and Mzb1; 2 T cell cluster (C2 and C9) expressed Trac and Tbrc2; 4 myeloid cells clusters (C3, C4, C7, and C12) expressed Itgam (encoding CD11B), Cd14, Ccl7, and Cxcl2. We next analyzed the difference of cell component between WT and *Il17b* ^-/-^ colitic mice. The results showed an obvious increase of cell percentage of cluster 4 (myeloid cells) and a decrease of cluster 7 of myeloid cells in *Il17b* ^-/-^ colitis mice compared to WT colitis mice (**Figures 4D, E**) and the trend was similar in two separate samples in each group (**Figure 4D**).

### IL17B deficiency increases neutrophils infiltration in colon lamina propria in colitis

**Figure 5:**
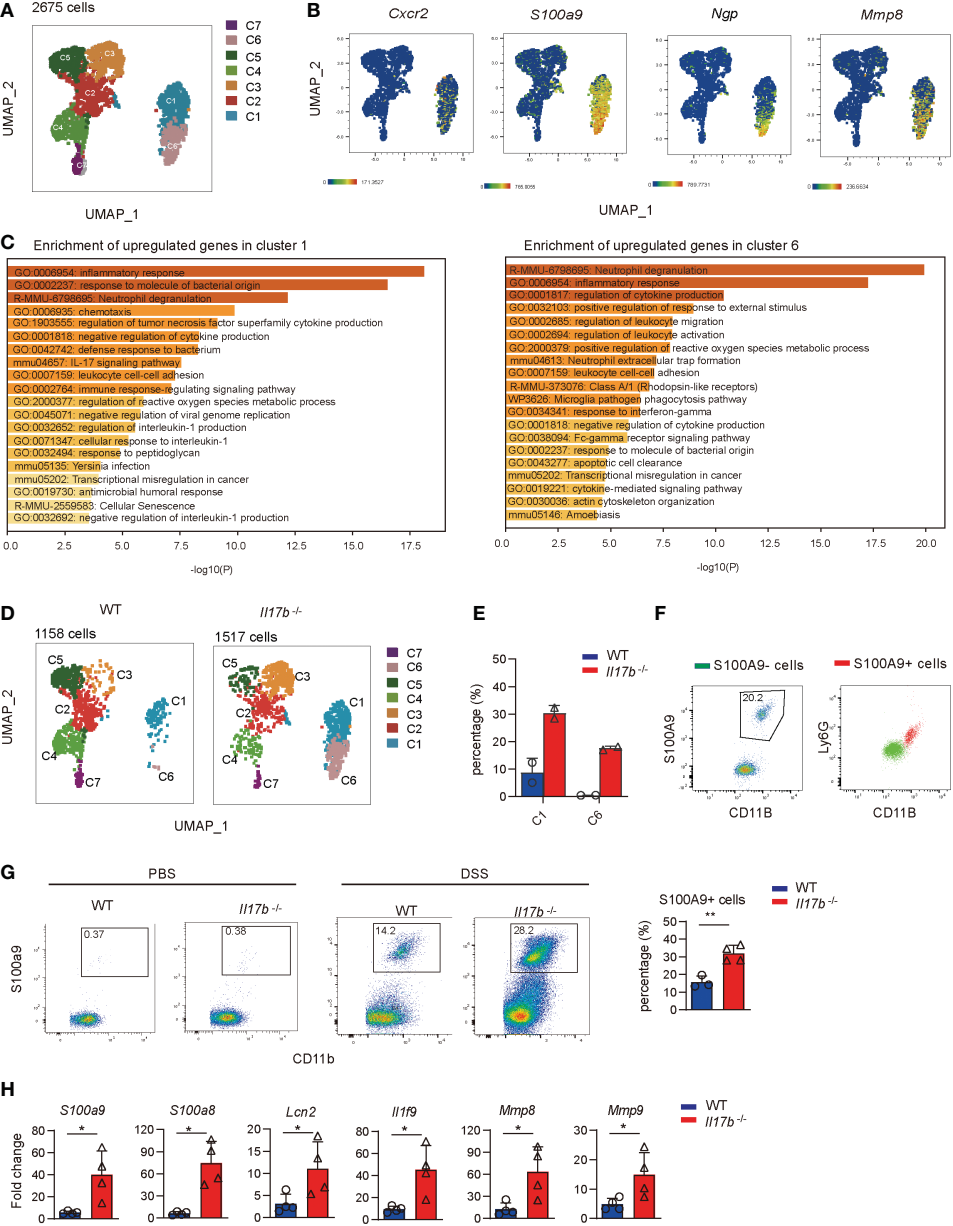
ScRNAseq analysis of myeloid cell response. **(A)** UMAP plot shows myeloid cell clusters. **(B)** The specific cell markers of neutrophils were projected on UMAP. **(C)** Gene enrichments of upregulated DEGs in cluster 1 and cluster 6. Top 20 of terms were shown. **(D)** UMAPs plots showing the difference of cell clusters distribution in WT and *Il17b* ^-/-^ colitic mice. **(E)** Percentage of cell cluster 1 and 6 in WT and *Il17b* ^-/-^ colitic mice. **(F–H)** WT and *Il17b* ^-/-^ mice (n = 4/group) were fed with 2.5% DSS water for 6 days. **(F)** The infiltration of CD11B^+^ S100A9^+^ in colon lamina propria was detected by flow cytometry on day 8 (right panel). The CD11B^+^ S100A9^+^ cells express Ly6G, confirming neutrophils phenotype (left panel). **(G)** The percentages of CD11B^+^ S100A9^+^ neutrophils in CD11B^+^ cells were compared between WT and *Il17b* ^-/-^ colitis mice. Bar graph showing the statistic of the CD11B^+^ S100A9^+^ cells in colitis mice. **(H)** The inflammatory genes expression in colonic lamina propria CD45^+^ cells was detected by real-time PCR. For bar graphs, data are expressed as mean ± SD. *P < 0.05; **P < 0.01, determined by unpaired *t* test. The experiment was performed 3 times.

We next focused on myeloid cell responses and reclustered them to 7 clusters (**Figure 5A**). Cluster 1 and cluster 6 were identified as neutrophils by exclusively expressing neutrophil markers Cxcr2, calprotectin (S100a9 and S100a8), inflammatory genes (Il1f9, Lcn2, Mmp8, and Retnlg), and neutrophilic granule protein (ngp) (**Figure 5B** and **Supplementary Table 5**). Gene enrichment further showed that signature genes in cluster 1 and cluster 6 were enriched into neutrophil activity and “inflammatory response’’, supporting their neutrophil feature and inflammatory characteristics (**Figure 5C**). Comparing cell component indicated neutrophils (C1 and C6) were significantly increased in *Il17b* ^-/-^ colitic mice (**Figures 5D, E**). Next we validated the scRNAseq results by detecting the signature marker of S100A9 and neutrophil markers Ly6G using flow cytometry. We identified CD11B^+^S100A9^+^ cells in colon tissue of DSS-induced colitis mice and they exclusively expressed in neutrophils (CD11B^+^Ly6G^high^ cells) (**Figure 5F**). Under steady state, there were few CD11B^+^S100A9^+^ neutrophils in colonic lamina propria in both WT and *Il17b* ^-/-^ mice (**Figure 5G**). In colitis, CD11B^+^S100A9^+^ neutrophils were significantly increased in WT mice compared to mice without DSS treatment, and this population was 2-fold increase in *Il17b* ^-/-^ colitic mice (**Figure 5G**). Further, we validated DEGs from scRNAseq in colon lamina propria CD45^+^ cells by real-time PCR. The expression of S100a9, S100a8, Lcn2, Il1f9, Mmp8, and Mmp9 were significantly increased in CD45^+^ cells from *Il17b* ^-/-^ colitis mice (*P* < 0.05, **Figure 5H**). Since scRNAseq discovered that these genes were exclusive expressed in neutrophils, the increased expression of these gens also verified the increased infiltration of neutrophils. Thus, these results indicate that CD11B^+^S100A9^+^neutrophils play important role in colitis and IL17B might negatively regulate these cells infiltration.

### Lack of IL17B causes increased inflammatory response in colonic macrophages

**Figure 6:**
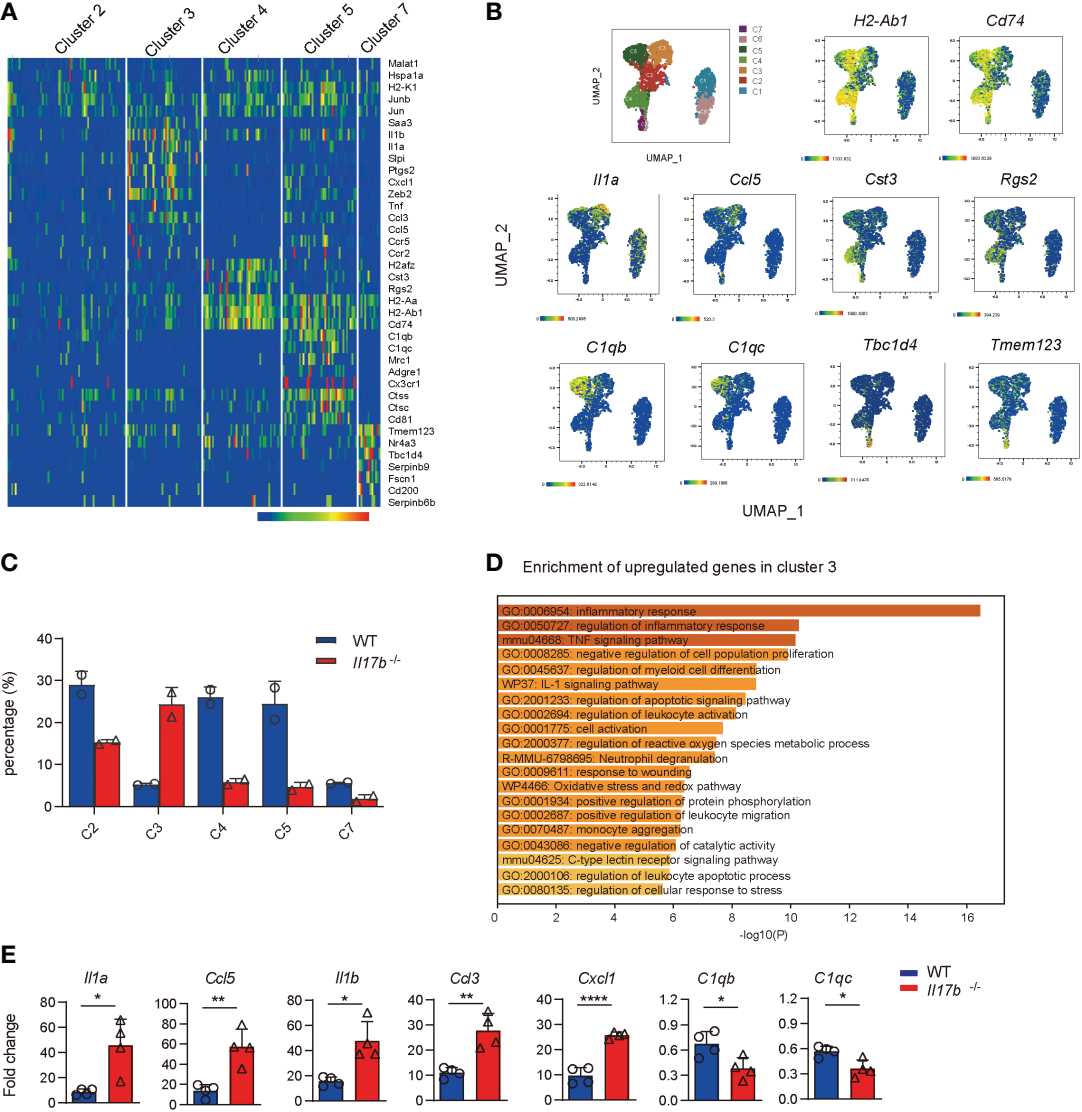
Increased inflammatory response in colonic macrophages in *Il17b* ^-/-^ colitic mice. **(A)** The heatmap shows the upregulated signature genes in colonic macrophages. **(B)** UMAP plots displaying marker genes expression for cell clusters. **(C)** Percentage of cell clusters of colonic macrophagse in WT and *Il17b* ^-/-^ colitic mice. **(D)** Gene enrichment of upregulated DEGs in cluster 3 of colonic macrophages. **(E)** The mRNA expression of some DEGs in colon lamina propria CD45^+^ cells of *Il17b* ^-/-^ and WT colitis mice were detected by real-time PCR. n = 7 for WT group and 6 for *Il17b* ^-/-^ mice. Data are expressed as mean ± SD. *P < 0.05, **P < 0.01, ****P < 0.0001, determined by unpaired *t* test.

Cluster 2, 3, 4, 5, and 7 were recognized as colonic macrophages. Cluster 3 was more like inflammatory macrophages expressing inflammatory cytokines Il1a, Ccl5, Saa3, Tnf, and Cxcl1 (**Figures 6A, B**, and **Supplementary Table 5**). Cluster 5 was colon resident macrophages with a distinct noninflammatory gene profile by expressing the classic complement genes (C1qb and C1qc), Cx3cr1, macrophage mannose receptor *Mrc1* (encoding CD206), *Adgre1* (F4/80), and lacking of pro-inflammatory genes (**Figures 6A, B**). Cluster 4 highly expressed Cst3 and Rgs2 (**Figures 6A, B**). Comparing cell component indicated the cluster 3 defined as inflammatory macrophages were significantly increased in *Il17b* ^-/-^ colitic mice (**Figure 6C**). Gene enrichment of the upregulated genes in cluster 3 also verified the inflammatory feature (**Figure 6D**). Further, we detected some cluster 3 specific genes in colon lamina propria CD45^+^ cells by real-time PCR. The expression of Il1a, Ccl5, Il1b, Ccl3, and Cxcl1 were significantly elevated in CD45^+^ cells from *Il17b* ^-/-^ colitis mice (P < 0.05, **Figure 6E**). Whereas resident macrophage marker C1qb and C1qc were reduced in CD45^+^ cells from *Il17b* ^-/-^ colitis mice (P < 0.05, **Figure 6E**). Collectively, these data suggest that deficiency of IL17B reshaped the population of colonic macrophages to a proinflammatory status.

### IL17B reduces LPS-stimulated inflammatory response

**Figure 7:**
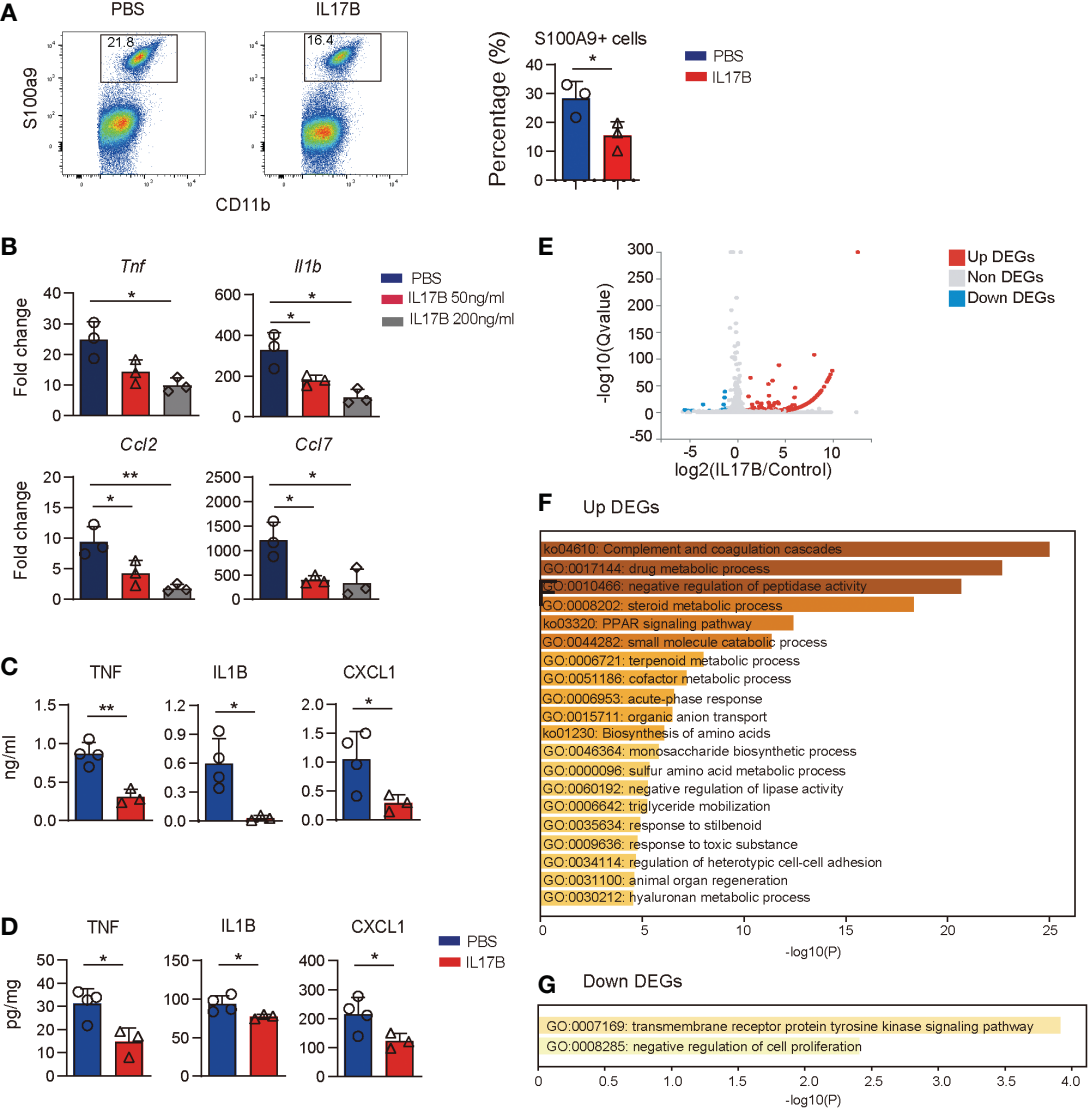
Treatment of IL17B reduces LPS-induced inflammation. **(A)** *Il17b* ^-/-^ mice were induced colitis and treated with IL17B as shown in figure 3. The infiltration of CD11B^+^ S100A9^+^ neutrophils in colon lamina propria was detected by flow cytometry. Bar graph showing the statistic of the CD11B^+^ S100A9^+^ cells in colitis mice. **(B)** BMDM was treated with LPS and IL17B for 2 hours (n = 3). The cytokines and chemokines expression of cells were detected by real-time PCR. **(C, D)** Mice were I.P. treated with LPS (n = 4) and IL17B (n = 3) for 2 hours and the inflammatory responses in serum **(C)** and colon tissue **(D)** were analyzed by ELISA. Data are mean ± SD. Statistics in **(B)** was done by ordinary one-way ANOVA with Turkey`s multiple comparisons test. Statistics in **(C)** and **(D)** was done with unpaired t test. *P < 0.05; **P < 0.01. **(E)** Volcano plot showing the DEGs of BMDM treated with IL17B detected by bulk RNAseq (triplicate samples for each treatment). **(F)** Gene enrichment of upregulated DEGs of IL17B-treated BMDM. **(G)** Gene enrichment of downregulated DEGs of IL17B-treated BMDM.

Since colonic resident macrophages are important for gut homeostatis, we hypothesize that IL17B can act on macrophages to regulate inflammatory response, then recruiting neutrophils. First, we treated *Il17b* ^-/-^ colitis mice with recombinant mouse IL17B and detected cell response in colon with flow cytometry and found that IL17B treatment can inhibit S100a9^+^ neutrophils infiltration in colon tissue (**Figure 7A**). Next, we tested whether IL17B could directly inhibit inflammatory response of macrophages. We stimulated bone marrow derived macrophages (BMDM) with LPS plus IL17B. The results showed that IL17B significantly inhibited the production of LPS-induced Tnf, Il1b, Ccl2, and Ccl7 in BMDM (**Figure 7B**). Further we detected the inhibitory role of IL17B on LPS-induced inflammation in vivo. Also IL17B significantly reduced LPS-induced TNF, IL1B, and CXCL1 production in serum (**Figure 7C**) and colon (**Figure 7D**), suggesting that IL17B could also inhibit inflammatory response in LPS-induced inflammation.

To explore the effect of IL17B on macrophages, we did bulk RNAseq of BMDM stimulated with IL17B. The results showed that there were 163 DEGs between IL17B vs. PBS-treated cells, with 146 upregulated DEGs and 17 downregulated DEGs (**Figure 7E** and **Supplementary Table 6**). The upregulated DEGs were enriched to “complement and coagulation cascades”, “negative regulation of peptidase activity”, “negative regulation of lipase activity”, and multiple metabolic process (**Figure 7F**). The downregulated DEGs were enriched to “transmembrane receptor protein tyrosine kinase signaling pathway” and “negative regulation of cell proliferaton” (**Figure 7G**). These results suggest that the inhibitory role of IL17B might go through negative regulating tyrosine kinase pathway and metabolic process.

## Discussion

IL17B is a member of IL17 cytokine family and its function is less characterized. In this study, we use scRNAseq to reveal that IL17B can exert its inhibitory role by regulating colon myeloid cell function in colitis, which is a new mechanism of IL17B function in colitis.

Currently, the function of IL17B has been interpreted differently. Initially, IL17B was cloned and defined as proinflammtory cytokine, since it can stimulate TNF and IL1B release from the monocytic cell line THP-1, and I.P. injection of IL17B caused marked neutrophil migration in normal mice (10, 11). IL17B has been reported to have a proinflammatory activity similar to IL25 in lung fibrosis (12) and eliciting type 2 cytokine responses (13). On other hand, it has been reported that IL17B exhibits a suppressive role in colitis and *Citrobacter rodentium* infection (6). Consistent with this study, our results also confirm the protective role of IL17B in colitis model using a different deletion system. The loss of IL17B in mice caused more severe DSS-induced colitis than WT mice and reconstitution of IL17B in *Il17b* ^-/-^ mice alleviated severity of colitis. Moreover, IL17B can inhibit LPS-induced inflammation in BMDM and in vivo sepsis model. Also treatment of BMDM with IL17B did not elevate proinflammatory cytokine response and increase genes expression by negative regulating cell response. All these results indicate that IL17B has an inhibitory effect in inflammation. The different reported function of IL17B might due to the different local environment and disease models used, suggesting that IL17B is a multifaceted cytokine.

Previous study shows that IL17B can compete with IL25 for binding IL17RB to inhibit the pathologic role of IL25 in colitis (6). However, whether there are other mechanisms of IL17B function in colitis is still unknown. Dysregulation of immune cell including the neutrophils, macrophages, dendritic cells, plasma cells, and T cells is important for IBD pathogenesis (3, 14), so we tried to explore the effect of IL17B on colon immune responses. We used scRNAseq to analyze colonic lamina propria immune response in colitis with an unbiased pattern, which provides a comprehensive view of immune response regulated by IL17B and avoids the limitation of studying a specific cell population. ScRNAseq analysis of CD45^+^ immune cells from colonic lamina propria of 2 WT and 2 *Il17b* ^-/-^ mice with colitis revealed the infiltration of myeloid cell, T cells, B cells, and plasma cells. The immune cell composition in colitis mice model is consistent with the recent reports of cell atlas of human IBD detected with scRNseq or CyTOF (15–17). Comparing the cell composition of colitis in *Il17b* ^-/-^ and WT mice revealed neutrophils were significantly increased and macrophages presented a proinflammatory status in colon tissue. Under normal conditions, there are few neutrophils in colon mucosa. In UC patients, unrestricted neutrophil infiltration and activation is associated with pathological changes in the gut and worse clinical outcomes (18). Neutrophils can potently affect inflammatory conditions by secreting or modifying pro-inflammatory cytokines, chemokines. In this study, neutrophils were characterized by expression of a series of inflammatory markers, such as *S100a9, S100a8, Il1f9, Mmp8, Mmp9, Tnf, Il1b*, and *Il1a*, which were verified by real-time PCR. Increased accumulation of CD11B^+^Ly6G^+^S100A9^+^ neutrophils in colon lamina propria in *Il17b* ^-/-^ colitis mice were validated by flow cytometry. S100A9 is Ca^2+^ binding protein belonging to the S100 family and an important pro-inflammatory mediator in acute and chronic inflammation. The S100A8 and S100A9 mRNA are differentially expressed in blood leucocytes of IBD patients compared to healthy control (19). Inhibition of S100A9 by a neutralizing anti-S100A9 antibody can reduce the colitis severity (20). Thus, S100A9 is a good biomarker and therapeutic target for colitis (19, 21). In this study, S100A9 is exclusive expressed in neutrophils, suggesting it is also a good biomarker of neutrophils (19, 21). Thus, IL17B deficiency results in increased neutrophils infiltration in response to colitis, which is a new function of IL17B.

However, how IL17B exerts its inhibitory role on myeloid cell is still unknown. It has been known that intestinal macrophages are important for maintaining gut homeostasis (22). Normally, intestinal macrophages express inhibitory genes and exhibit non-inflammatory genes profile, which play a role in polarization of myeloid cell. Intestinal macrophages can negatively regulate neutrophil infiltration during colitis (23, 24). The depletion of intestinal mononuclear phagocytes (macrophages and dendritic cells) has been reported to increase neutrophil infiltration and increase the severity of injury in the DSS-induced colitis model (24). We propose that IL17B might act on intestinal macrophages to inhibit chemokine production, thereby inhibiting neutrophil infiltration. ScRNAseq reveals that intestinal macrophages in *Il17b* ^-/-^ mice shifted from non-inflammatory population into proinflammatory population (**Figure 6**), which supports our hypothesis. We also test effect of IL17B on BMDM showing that IL17B can inhibit LPS-induced inflammatory cytokine and chemokine release in both BMDM and *in vivo* LPS model, suggesting its directly inhibitory effect on macrophages. However, *in vivo*, whether the proinflammtory macrophages is converted from intestinal resident macrophages or derived from recruiting monocytes remains to be illustrated. Also, the detailed mechanisms and signaling related to the inhibitory function need to be further illustrated. Overall, we defined a new function of IL17B by regulating intestinal macrophages and recruiting neutrophils in colitis. However, detailed mechanisms of how IL17B regulates macrophage functions and neutrophils infiltration will be further explored.

In this study, we focus on the effect of IL17B on immune cell responses in colitis model. Besides immune dysregulation, other factors, such as barrier function of gut mucosa, microbiota, and gut metabolism, are also important for the pathogenesis of colitis. Whether IL17B will affect these factors needs further investigation.

As for IBD, inflammatory response in colonic mucosa is a hallmark of the disease pathogenesis.As for the process of IBD, proinflammatory cytokines and anti-inflammatory cytokines are induced simultaneously and their balance is important for the progress of disease. IL10 has been induced in IBD patient and provides an anti-inflammatory function. Cytokine targeted therapies offer amelioration of inflammation (25). TNF and IL6 have been extensively defined as risk factors for IBD. Anti-inflammatory drug such as anti-TNF antibodies have been tried clinically with great success. Another option is using anti-inflammatory cytokines. Currently, several cytokines like IL10 (26, 27), IL27 (28), IL35 (29), IL37 (30, 31), and TGFB are defined as inhibitory cytokine and can protect colitis. Mucosal delivery of the immunosuppressive cytokine, such as IL27, IL35, can reduce colitis (28, 29). Injection of recombinant IL17B can reduce colitis severity, suggesting that IL17B is another novel anti-inflammatory cytokines, which might have the therapeutic potential for IBD patients.

## Data availability statement

The datasets presented in this study can be found in online repositories. The names of the repository/repositories and accession number(s) can be found below: https://www.ncbi.nlm.nih.gov/geo/query/acc.cgi?acc=GSE161987, https://www.ncbi.nlm.nih.gov/geo/query/acc.cgi?acc=GSE162062.

## Ethics statement

The studies involving human participants were reviewed and approved by the Ethics Committee at Chongqing General Hospital. The patients/participants provided their written informed consent to participate in this study. The animal study was reviewed and approved by the Institutional Animal Care and Treatment Committee of West China Hospital, Sichuan University.

## Author contributions

XiaomZ, Xiaokz and XS performed most of the experiments and analyzed data. XiaokZ generated and bred *Il17b* ^-/-^ mice. XZ and CX performed scRNAseq experiment. CH, YX, NW helped preparing the experiment materials. YZ, GG, WZ, YL, and KL assisted with the experiments and provided technical support. QZ supervised the project and did critical revision of manuscript. HG provided the patient samples and supervised the study of clinical samples and revised the manuscript. YS obtained funding, designed the study and prepared the manuscript. All authors contributed to the article and approved the submitted version.
