# Supplementary figures and images for: Interleukin 17 B regulates colonic myeloid cell infiltration in a mouse model of DSS-induced colitis
Source: Front Immunol. 2023 Feb 6;14:1055256. doi: 10.3389/fimmu.2023.1055256 (PMC9940313; doi:10.3389/fimmu.2023.1055256)

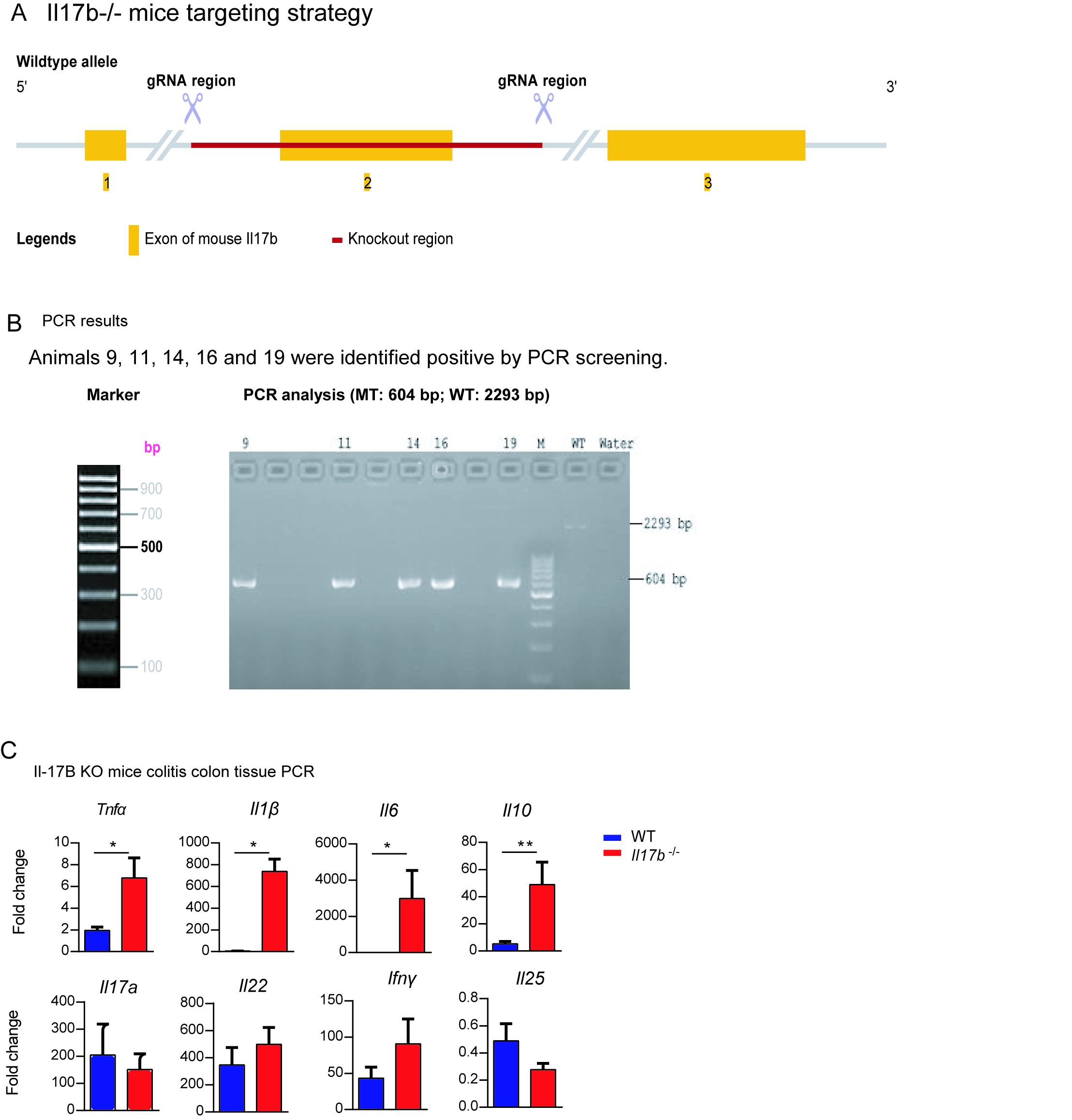

Supplement: Supplementary file 4 [file Image_1.tif]

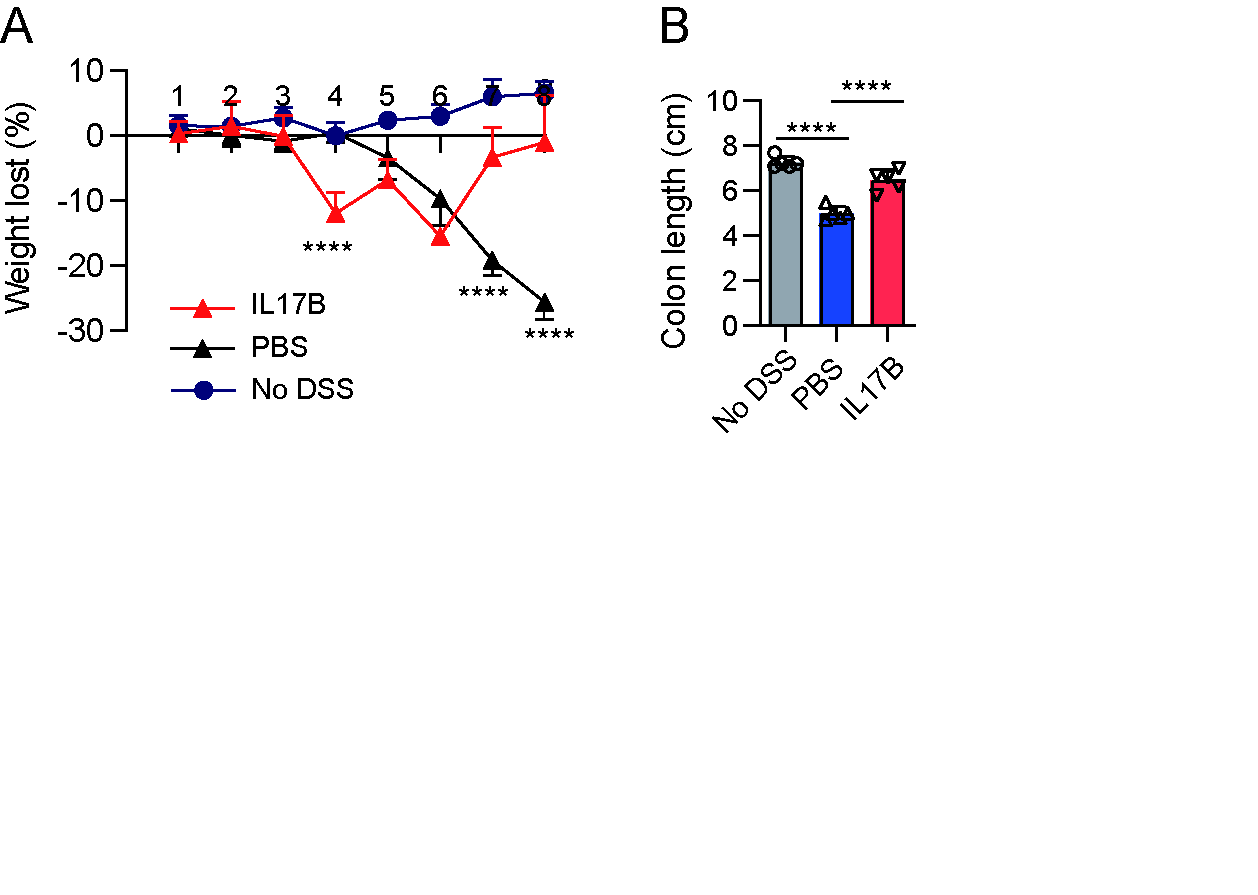

Supplement: Supplementary file 5 [file Image_2.tif]

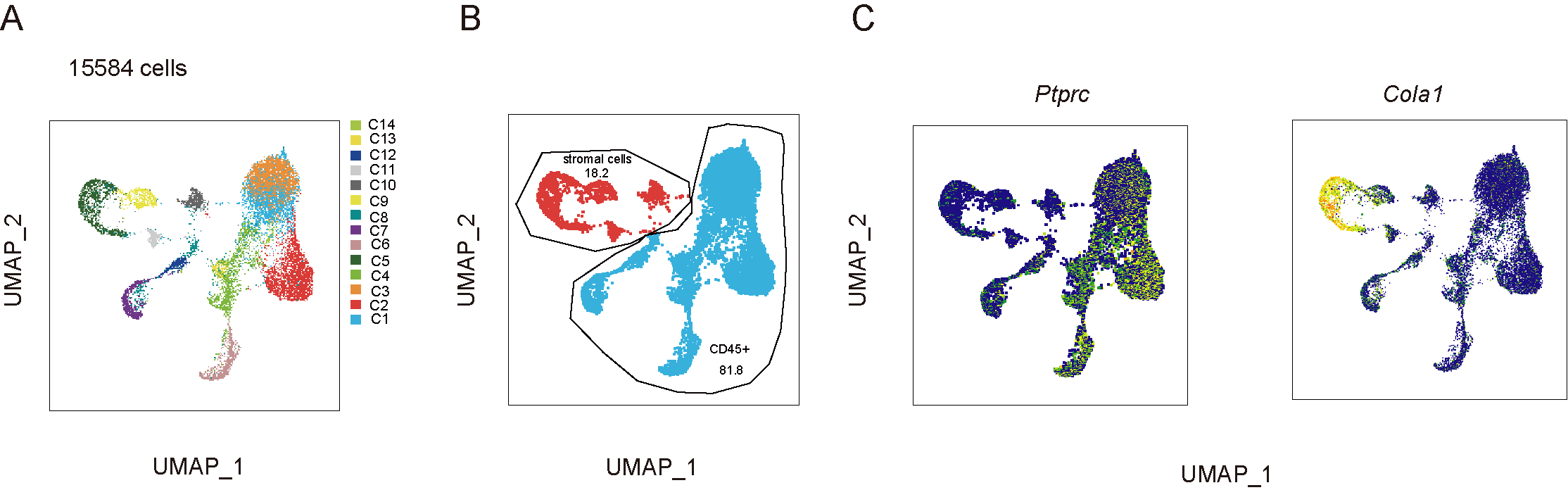

Supplement: Supplementary file 6 [file Image_3.tif]
