# Supplementary material for: Interleukin 17 B regulates colonic myeloid cell infiltration in a mouse model of DSS-induced colitis
Source: Front Immunol. 2023 Feb 6;14:1055256. doi: 10.3389/fimmu.2023.1055256 (PMC9940313; doi:10.3389/fimmu.2023.1055256)
Supplement: Supplementary file 7 [file Table_1.docx]

Supplementary Table 1: Clinical characteristic of IBD patients

| Variables | No. of patients |
| --- | --- |
| Gender (Female/Male) | 6/17 |
| Age (years; median, range) | 32,17-56 |
| Smokers (yes/no) | 8/15 |
| **Disease type** |  |
| UC | 10 |
| CD | 13 |
| **Disease level** |  |
| Active |  |
| Mild | 6 |
| Moderate | 6 |
| Severe | 3 |
| Remission | 8 |
