# Supplementary material for: Interleukin 17 B regulates colonic myeloid cell infiltration in a mouse model of DSS-induced colitis
Source: Front Immunol. 2023 Feb 6;14:1055256. doi: 10.3389/fimmu.2023.1055256 (PMC9940313; doi:10.3389/fimmu.2023.1055256)
Supplement: Supplementary file 8 [file Table_2.docx]

**Supplementary Table 2. Primers used in real-time PCR.**

| Primer name | Primer sequence |
| --- | --- |
| *Il6*- Forward | 5′-CCTCTCTGCAAGAGACTTCC-3′ |
| *Il6-* Reverse | 5′-CTCCGGACTTGTGAAGTAGG-3’ |
| *Il1a*- Forward | 5’-CGAAGACTACAGTTCTGCCATT-3’ |
| *Il1a-* Reverse | 5’-GACGTTTCAGAGGTTCTCAGAG-3’ |
| *Il1b*- Forward | 5’-GGACCCCAAAAGATGAAGGGCTGC-3’ |
| *Il1b* - Reverse | 5’-GCTCTTGTTGATGTGCTGCTGCG-3’ |
| *Tnf*- Forwad | 5’-CCTATGTCTCAGCCTCTTCTCAT-3’ |
| *Tnf-* Reverse | 5’-CACTTGGTGGTTTGCTACGA-3’ |
| *C1qb*- Forward | 5’-CCAGGTCATTCGCTTCGAAAA-3’ |
| *C1qb* - Reverse | 5’-GAGGCCAGGCACCTTGCA-3’ |
| *C1qc* - Forward | 5’-CAACGCCCTCGTCAGGTT-3’ |
| *C1qc* -Reverse | 5’-ACTTCCCTGTGCTTGGGTTGT-3’ |
| *Cxcl1*-Forward | 5’-ATGGCTGGGATTCACCTCAA-3’ |
| *Cxcl1*--Reverse | 5’-AGTGTGGCTATGACTTCGGT-3’ |
| *Ccl5-*Forward | 5’-AGATCTCTGCAGCTGCCCTCA-3’ |
| *Ccl5*-Reverse | 5’-GGAGCACTTGCTGCTGGTGTAG-3’ |
| *Ccl2*- Forward | 5’-TTAAAAACCTGGATCGGAACCAA-3’ |
| *Ccl2*- Reverse | 5’-GCATTAGCTTCAGATTTACGGGT-3’ |
| *Ccl7-* Forward | 5’- CTC ATA GCC GCT GCT TTC AGC ATC-3’ |
| *Ccl7*- Reverse | 5’- GTC TAA GTA TGC TAT AGC CTC CTC-3’ |
| *Mmp8*- Forward | 5’-TCAACCAGGCCAAGGTATTG-3’ |
| *Mmp8*-Reverse | 5’-ATGAGCAGCCACGAGAAATAG-3’ |
| *Mmp9*- Forward | 5’-TTGGTTTCTGCCCTAGTGAGAGA-3’ |
| *Mmp9*- Forward | 5’-AAAGATGAACGGGAACACACAGG-3’ |
| *S100a8*- Forward | 5’-ACAAGGAAATCACCATGCCCT-3’ |
| *S100a8*- Reverse | 5’-TGTGAGATGCCACACCCACT-3’ |
| *S100a9*- Forward | 5’-CCACCATCATCGACACCTTCCATC-3’ |
| *S100a9*- Reverse | 5’-AGGTTGCCAACTGTGCTTCCAC-3’ |
| *Lcn2*- Forward | 5’-AAGGAACGTTTCACCCGCTT-3’ |
| *Lcn2*- Reverse | 5’-AATGCATTGGTCGGTGGGGA-3’ |
| *Il1f9*- Forward | 5’-GGGGAGGTTTTTGACTTGGAC-3’ |
| *Il1f9*- Reverse | 5’-TACTCTGTGGCTTCGTGGAACT-3’ |
| *Actb*- Forward | 5’-GGCTGTATTCCCCTCCATCG-3’ |
| *Actb* - Reverse | 5’-CCAGTTGGTAACAATGCCATGT-3’ |
| *Il22*- Forward | 5’- CATGCAGGAGGTGGTACCTT-3’ |
| *Il22*- Reverse | 5’- CAGACGCAAGCATTTCTCAG-3’ |
| *Il17*- Forward | 5’- CTCCAGAAGGCCCTCAGACTAC-3’ |
| *Il17*- Reverse | 5’- GGGTCTTCATTGCGGTGG-3’ |
| *Ifng*- Forward | 5’- GATGCATTCATGAGTATTGCCAAGT-3’ |
| *Ifng* - Reverse | 5’- GTGGACCACTCGGATGAGCTC-3’ |
| *Il25*- Forward | 5’- TTGGAGCTATGAGTTGGACAGGGA-3’ |
| *Il25*- Reverse | 5’- AGACCGTCTGGTTGTGGTAAAGTG-3’ |
| *hIL17B-*Forward  *hIL17B-*Reverse  *hGAPDH*-Forward  *hGAPDH-*Reverse | *ATGTCGCTCGTGCTGCTAAG*  *AGCCACATTGAACGGTCGG*  GCAAATTCCATGGCACCGT  GCCCCACTTGATTTTGGAGG |
